# Impact of traditional culture on *Camellia reticulata* in Yunnan, China

**DOI:** 10.1186/s13002-015-0059-6

**Published:** 2015-10-22

**Authors:** Tong Xin, Jan de Riek, Huijun Guo, Devra Jarvis, Lijuan Ma, Chunlin Long

**Affiliations:** College of Life and Environmental Sciences, Minzu University of China, Beijing, 100081 China; Plant Sciences Unit, Institute for Agricultural and Fisheries Research, Melle, 9090, Belgium; Department of Forestry, Yunnan People’s Government, Kunming, 650010 China; Bioversity International, Via dei Tre Denari 472/a, 00057 Maccarese, Rome Italy; Kunming Institute of Botany, Chinese Academy of Sciences, Kunming, 650201 China

**Keywords:** Ethnobotany, *Camellia reticulata*, *Cha-Hua*, Traditional culture, Biodiversity

## Abstract

**Background:**

*Cha-hua* (*Camellia reticulata*) is one of China’s traditional ornamental flowers developed by the local people of Yunnan Province. Today, more than 500 cultivars and hybrids are recognized. Many ancient camellia trees still survive and are managed by local peopl. A few records on *cha-hua* culture exist, but no studies expound the interaction between *C. reticulata* and traditional culture of ethnic groups. The contribution of traditional culture of different nationalities and regions to the diversity of *Camellia reticulate* is discussed.

**Methods:**

Ethnobotanical surveys were conducted throughout Central and Western Yunnan to investigate and document the traditional culture related to *Camellia reticulata*. Five sites were selected to carry out the field investigation. Information was collected using participatory observation, semi-structured interviews, key informant interviews, focus group discussions, and participatory rural appraisal (PRA).

**Results:**

Most of the ancient camellia trees were preserved or saved in the courtyards of old buildings and cultural or religious sites. Religion-associated culture plays an important role in *C. reticulata* protection. In every site we investigated, we found extensive traditional culture on *C. reticulata* and its management. These traditional cultures have not only protected the germplasm resources of *C. reticulata*, but also improved the diversity of *Camellia* cultivars.

**Conclusions:**

There are abundant and diverse genetic resources of *cha-hua*, *Camellia reticulata* in Yunnan. *Cha-hua* is not only an ornamental flower but also has been endowed with rich spiritual connotation. The influence of traditional culture had improved the introduction and domestication of wild plants, breeding and selection of different varieties, and the propagation and dissemination of the tree in Yunnan. However, either some ancient *cha-hua* trees or their associated traditional culture are facing various threats. The old *cha-hua* trees and the ethnic camellia culture should be respected and protected since they have made great contributions in the history, and will make more contributions in the future.

## Background

Yunnan Province, with its geographical location, complicated landscapes, various climate conditions, and numerous indigenous ethnic groups, is recognized as the richest region in biocultural diversity in China [1, 2]. Throughout history, people have interacted with their natural environment in multiple ways shaping human the structure of human society, through the utilization of natural resources for subsistence and commercial objectives [3, 4], for example [5]. This rich biodiversity and cultural diversity forms a part of the daily routine, social customs, needs, food habits, ailments, and notions about natural phenomena [6]. Faith tradition, taboos and cultural association with plant species have helped in the conservation of plant diversity, which can be studied from an ethnobotanical perspective [3].

In Chinese, *cha-hua* refers to the ornamental trees of genus *Camellia* in the Theaceae family [7, 8]. China is regarded as the origin and distribution center of *Camellia,* with 97 species, in which 76 species are endemic to the country [7–9]. The genus *Camellia* is normally divided into five categories. *Camellia reticutala* Lindl. and its close relatives represent an important group, mostly distributed in Yunnan Province. Camellias are considered in Yunnan to have great economic values. Some are extremely important flowering ornamentals and oil-bearing sources with numerous cultivars [10, 11].

In Yunnan Province, *cha-hua* is the most common name especially used for *C. reticutala*. For the indigenous people of Yunnan, *cha-hua* trees have been part of their culture for generations, occupying all aspects of their lives [12]. This special relationship between the local people and the camellias has created a unique culture of the camellias in Yunnan. In many parts of Yunnan, especially in Central and Western Yunnan, *cha-hua* trees are widely cultivated in ancient temples, scenic spots, public and private gardens. There is overlapping of the distribution of *cha-hua* and the ethnic groups of Dali, Chuxiong, Lijiang, Tengchong and Kunming, together with the different cultures of Bai, Yi, Naxi, Han and other nationalities, among whom mutual cultural influences have co-existed for a long time.

According to historical records, *cha-hua* was cultivated or semi-cultivated as early as in the Sui and Tang dynasties (1500 years ago) [8, 13, 14]. The tree also appeared in many poems, inscriptions and other literature [13, 15]. During hundreds of years of cultivation, intra- and inter-specific hybridizations have occurred both naturally and artificially [16]. Through the centuries, the indigenous people of Yunnan have cultivated and appreciated camellias. The impact of traditional culture on *cha-hua* may be one of the major factors that has supported the conservation of the biological diversity of the species. Currently, more than 500 cultivars and hybrids of *cha-hua* have been recognized [17]. Ethnobotanical surveys can help to collect important information on the role of traditional culture in enhancing the genetic diversity and conserving *C. reticulata*.

Loss of biological resources, an increasingly globalized society, cultural homogenization and desire for modernization are major factors attributed to the general decline in cultural knowledge about plants, and the disappearance of traditional practices that involve these plants [18–21]. Integration of cultural and biological diversity is often left out of sustainable development plans [19]. Most focused on the maintenance of diversity of cultural species and not their use in sustainable development [22–25].

The investigation of the cultural values of plant species plays a significant role in modern medicine, farming, pharmaceutical and nutritional industrial sectors of a society [26, 27]. The exploration and record of cultural factors of plants are necessary and urgent if this information is to be integrated into sustainable agricultural development plans [28]. Few publications are attributed to the traditional knowledge or perceptions of the local folk and the management and use of camellias linked with local traditional cultural interrelationships. We conducted ethnobotanical surveys throughout the distribution area of *C. reticulata* in Yunnan Province to understand the impact of that traditional culture and ethnic diversity has had on the diversity and conservation of *C. reticulata*.

## Methods

### Study area

**Fig. 1:**
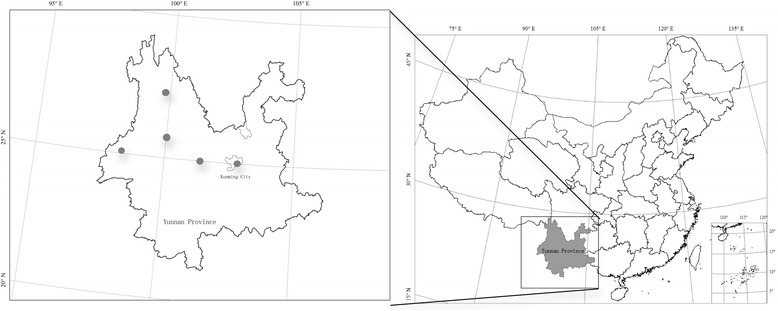
Location of study areas. Dots show the investigation sites. *Camellia reticulata* is mainly distributed in Central and Western Yunnan Province, Southwest China

The study was carried out in five areas of Yunnan Province: Kunming, Dali, Lijiang, Tengchong, and Chuxiong, located in Central and Western Yunnan Province (between 24°12’–26°86’ N and 98°13–102°42’ E) (Fig. 1) (Table 1).

**Table 1:** Sites to investigate *Camellia reticulata* in Central and Western Yunnan Province

Cities	Sites
Kunming	Kunming Botanical Garden; Heilongtan Park;
	Golden Temple Park;
	West Hill;
	Yiliang County
Dali	Yu’er Park; Chongsheng Temple and three Pagoda;
	Zhang Clan Garden;
	Dali Bai Autonomous Prefecture Museum
Lijiang	Yufeng Temple;
Tengchong	Camellia base; Laifeng Mountain; Longhua Temple; Hexie Village
Chuxiong	Zixi Mountain;
	E'lu Park

Kunming is the capital city of Yunnan, with a total area of 2143 km^2^ and a population of about 7.21 million. It is located in the low latitude plateau with an average elevation of 1900 m above sea level. Its annual rainfall is 924 mm with an average temperature of 16.5 °C. Kunming is also the provincial center with numerous diverse nationalities. Nine nationalities have lived in Kunming for a long time, i.e. Yi, Bai, Miao, Hui, Dai, Hani, Lishu, Zhuang, and Han.

Chuxiong Yi Autonomous Prefecture is located in the north of the central Yunnan plateau, with an area of 29,256 km^2^ and a population of about 2.684 million. Its average altitude is 1770 m above sea level, and the annual rainfall is 851 mm with an average temperature of 15.7 °C. The minority nationalities (non-Han Chinese) account for one third of the total population, in which the Yi ethnic group is the largest nationality [29].

Dali Bai Autonomous Prefecture is located in northwestern Yunnan, with an area of 29,459 km^2^ and a population of 3.456 million. Its average altitude is 2090 m above sea level, and the annual rainfall is 836 mm with an average temperature of 15.1 °C. Dali was the site of two kingdoms, the Dali Kingdom and Nanzhao Kingdom. It is one of the places where Yunnan culture originated. Majority is the Bai people, together with Yi, Naxi, Miao, Han and others.

Lijiang City is also located in northwest Yunnan, boarding on Sichuan Province. It is in a region where the Qinghai-Tibet Plateau and Yunnan-Guizhou Plateau converges. The area is 20,600 km^2^ and a population is 1.248 million. Owing to its lower latitude and higher elevation (2400 m), the city center of Lijiang experiences a mild subtropical highland climate with an average temperature of 12.6 °C. In Lijiang, there are 20 % of Naxi people, and the others are Yi, Bai, Lisu, Tibetan and Han. The Naxi’s Dongba culture is a representative of traditional culture in the region.

Tengchong is a county belonging to Baoshan City, west of Yunnan Province, situated at the southwestern end of the Hengduan Mountains (elevation varied from 930 to 3780 m). The county seat is 1640 m above sea level, surrounded by a group of young volcanoes, acclaimed as a “Natural Volcanic Geological Museum”, for it reflects the young volcano and terrestrial heat in the most concentrated, magnificent and typical manner. The area of Tengchong County is 5693 km^2^, and the population is 0.594 millions. There are different nationalities living in the country including Han, Yi, Dai and Lisu. Abundant plant resources are distributed in this area because of its special geographical location and climate diversity [30].

### Literature studies

Prior to fieldwork, relevant literature was consulted to obtain information on the local culture of areas with Camellia. This information was used in choosing the specific study sites. Literature reviews included searches with Google Scholar, PubMed, Scopus, Web of Science and the Chinese databases such as VIP and Wanfang.

### Field surveys

Ethnobotanical data were collected through different interview methods: participatory rural appraisal (PRA), participatory observation, semi-structured interviews, key informant interviews, focus group discussions and cultural anthropology [31–35]. Fieldwork was conducted from November to December 2012, and from January to February 2014.

Key informant interviews collected information from *Camellia* experts, scenic spot managers, private garden owners, *Camellia* enthusiasms, and visitors in *Camellia* gardens or temples with old *Camellia* trees. Old *Camellia* gardens, parks, and temples were visited as well. Particular attention was payed to collecting information of Buddhism culture related to *cha-hua*. In villages, semi-structured interviews and focus group discussions were predominantly used to obtain information. In total 120 people were interviewed, of which 77 were males and 43 were females. All of them were over 20 years old.

## Results and discussions

### Religion-associated culture of *Camellia reticulata*

#### Protected by Buddhism

The *cha-hu* (*C. reticulata*) has always been denoted as a plant that represents good fortune, and has been treated as a chastity flower in people’s mind. The chastity flowers are closely related to religion [15]. Many groups used beautiful flowers as sacrifices for worship, especially in the Buddha rituals. According to ancient records after the Yuan Dynasty (AD 1271–1368), *C. reticulata* became the Buddha flower [17]. Buddhism called *Camellia* as “Man-tuo-luo”. It is the auspicious flower for consecration when chanting the Buddha “Lotus Sutra”. The Buddhism monasteries planted *cha-hua* trees to decorate the temple scenery and to show sacred auspicious aura. The unique temperament and flower culture of *cha-hua* could meet the demands of Buddhist doctrine, and naturally became the best tree for Buddha.

**Fig. 2:**
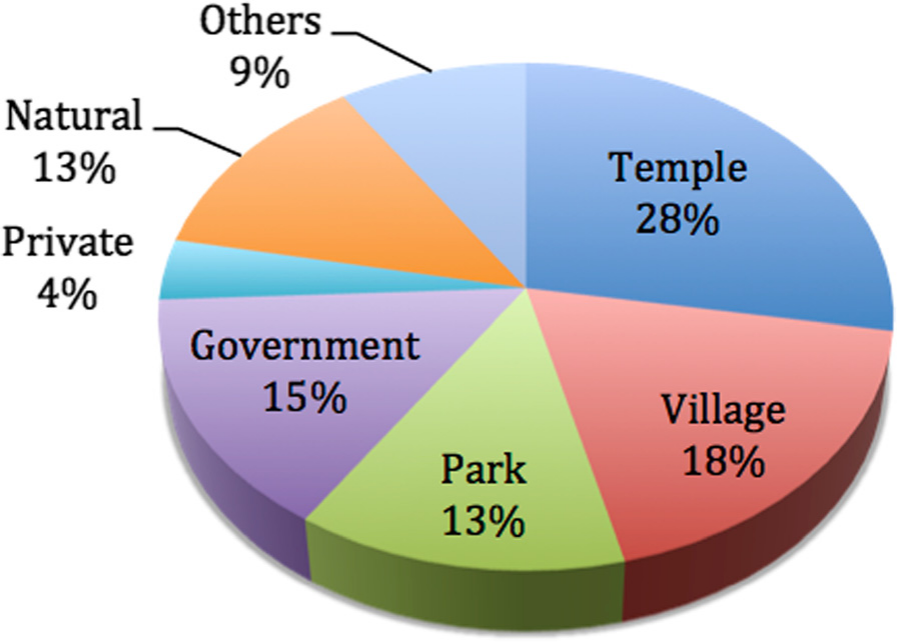
Ancient trees distribution in Kunming

**Fig. 3:**
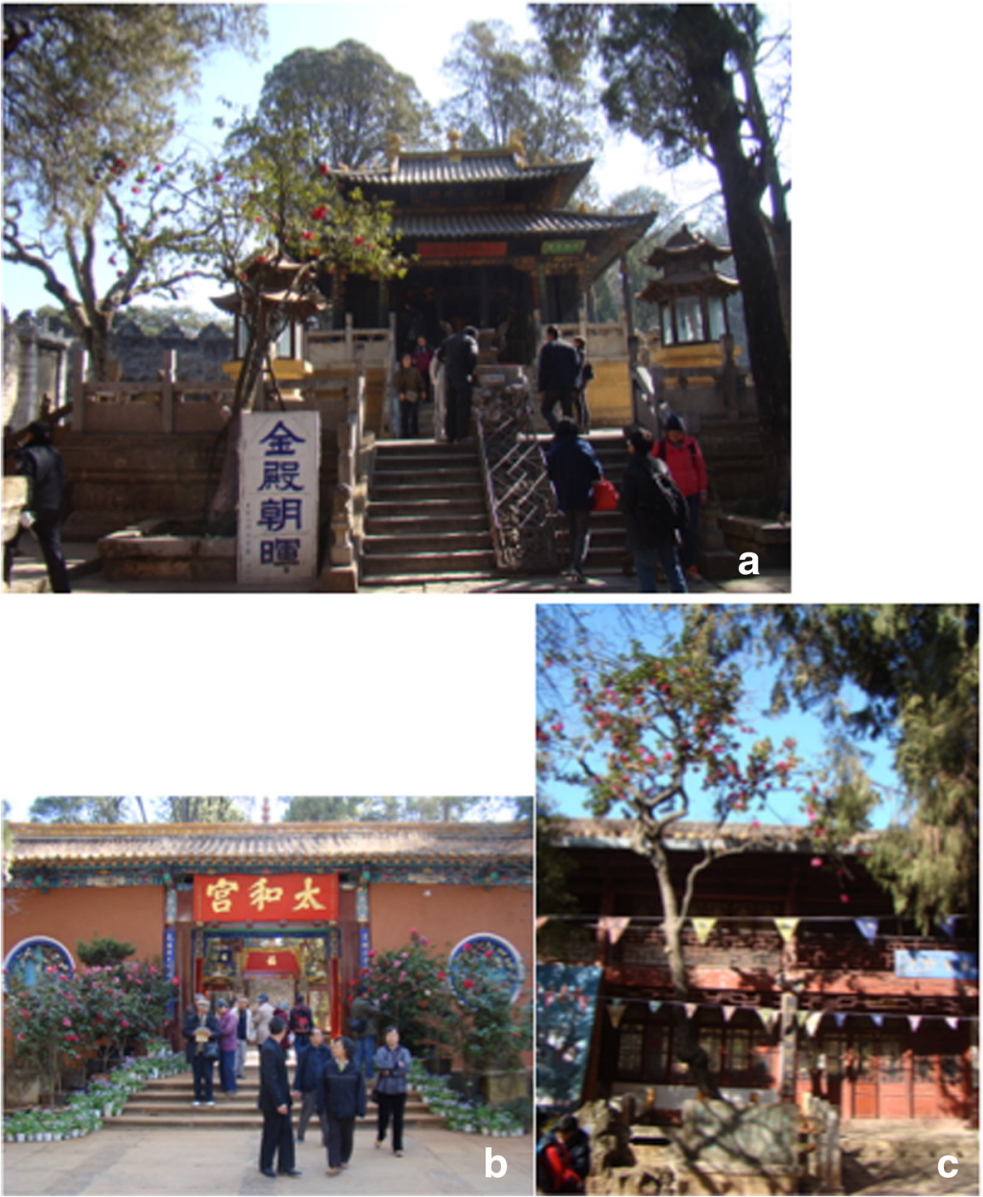
(**a** & **b**): Ancient *Camellia reticulata* ‘Shizitou’ in Jindian Park; **c**: Ancient *Camellia reticulata* ‘Zaotaohong’ in Heilongtan Park

According to our investigations, in the Kunming area, there are 206 ancient trees (or heritage trees) of *cha-hua*. Figure 2 shows that many of these ancient trees (28%) are maintained in old temples. Among them, 16 ancient camellia trees are maintained in good conditions (Table 2). Five ancient *cha-hua* trees were discovered in temples, occupying about one third of the total (Fig. 3). The Panlong temple, Huating Temple, Zixi Mountains and Jizu Mountains are famous Buddhist sites of Yunnan. The ancient *C. reticulata* trees have been well maintained in these shrines. Many ancient *Camellia* trees were found in Chuxiong’s Budhism temples and Taoism temples or their relics. In Zixishan Mountains there are many relics of temples. Of the 59 cultivated *Camellia* types (Table 3), 26 are distributed in the relic of temples (44.8 %).

**Table 2:** Ancient *Camellia reticulata* trees in Kunming

No.	Cultivar names	Age (year)	Growth	Conservation	Sites
1	‘Lion’s Head’	150	Good	None	Longtou Street, Northern Suburb of Kunming
2	‘Lion’s Head’	160	Health	General	Golden Temple Park
3	‘Early Crimson’	170	Good	General	Black Dragon Pool Park
4	‘Lion’s Head’	105	Health	Good	The Huating Temple, the largest Buddhist temple in Kunming, located in the Western Hills.
5	‘Pine Cone Scale’	650	Health	Good	Panlong Temple, Jinning
6	‘Lion’s Head’	160	Health	Good	Zhangfu Village
7	‘Early peony’	160	Health	General	Dongjia Village
8	‘Early peony’	160	Health	Good	Dajie Village
9	‘Lion’s Head’	210	Health	Good	Chijiu Town
10	‘Pine Cone Scale’	210	Health	Good	Chijiu Town
11	Jing’an Camellia	230	Health	General	Yiliang County, Jin’an Village
12	‘Lion’s Head’	310	Bad	None	Qidian Town
13	‘Lion’s Head’	500	Good	General	Songming County, Pijia Village
14	‘Early Crimson’	100	Health	Good	Songming County, Dianwei Town
15	‘Early Crimson’	300	Health	General	Songming County, Pijia Village
16	‘Lion’s Head’	400	General	None	Xundian County, Changchong Village

**Table 3:** Some ancient trees of *Camellia reticulata* in Chuxiong City

No.	Cultivar names	Latitude (N)	Longitude (E)	Elevation (m)	Location
1	‘Tongzimian’	24°59'24"	101°23'47"	1975	Mishi Temple of Hongqiang Village, Donghua Township
2	‘Zixi’ & ‘Tongzimian’	24°59'54"	101°26'03"	2260	Donglin Temple of Zixishan Mountain
3	‘Chuxiongcha’	24°59'22"	101°29'20"	1865	Lu’s Family Hall, Daluyi Village, Ziwu Township
4	‘Luchengchun’	25°03'03"	101°37'03"	1930	Zhuxichong of Zhuangdian, Donggua Township
5	‘Dalicha’	24°55'25"	116°30'48"	1880	Kuame Mishi Temple of Donghua Village, Donghua Township
6	‘Zehe’	24°57'09"	101°27'05"	1878	Xiaozehe Mishi Temple of Bendong Village, Donghua Township
7	*C. reticulata* f. *simplex*	24°57'21"	101°01'21"	1982	Shangxinfang Mishi Temple, Sanjie Township
8	‘Chuxiong-dalicha’	25°05'20"	101°33'20"	1880	Lijia Village of Dengguan of Donggua Township
9	‘Chudie’	25°02'54"	101°37'15"	1921	Zhuxichong of Zhuangdian, Donggua Township
10	‘Zehe’	24°56'47"	101°33' 46"	1849	Xiamafang Mishi Temple, Ziwu Township
11	‘Dalicha’	24°58'09"	101°38'06"	2057	Shuicaoqing Temple of Dadong Village, Lucheng Township
12	*C. reticulata* f. *simplex*	25°00'03"	101°24'52"	2344	Nianfotang of Zixishan Mountain
13	‘Donglin’	24°59'57"	101°25'03"	2301	Zhiguanglin Temple of Zixishan Mountain
14	‘Xiangguocha’	25°01'54"	101°23'48"	2354	Shisangcheng of Zixishan Mountain
15	‘Zhaoqing’	25°01'54"	101°23'42"	2350	Shisangcheng of Zixishan Mountain
16	*C. reticulata* f. *simplex*	24°59'58"	101°24'43"	2342	Xilin Temple of Zixishan Mountain
17	*C. reticulata* f. *simplex*	N25°00'20"	E101°25'13"	2482	Xilin Temple of Zixishan Mountain
18	‘Seben’	25°01'54"	101°23'48"	2354	Shisangcheng of Zixishan Mountain
19	‘Songzike’	25°00'51"	101°24'35"	2413	Dajing Temple of Longjing, Zixi Mountain
20	‘Shizitou’	25°05'46"	101°33'22"	1880	Lijia Village of Dengguan of Donggua Township
21	‘Chuxiong-dalicha’	25°05'45"	101°33'19.7"	1880	Lijia Village of Dengguan of Donggua Township
22	‘Guomei’	25°01'53"	101°26'15.4"	1898	Wangjiaju Mishi Temple, Zixi Township
23	*C. reticulata* f. *simplex*	25°01'33"	101°36'16.5"	1839	Gangeding of Lijia of fumin Village, Lucheng Township
24	‘Dalicha’	25°00'01"	101°24'60"	2339	Guzhulin of Zixishan Mountain
25	‘Lichan’	25°00'03"	101°24'52"	2339	Nianfotang of Zixishan Mountain
26	‘Zixia’	25°00'03"	101°24'52"	2339	Nianfotang of Zixishan Mountain
27	‘Lingfeng’	25°00'04"	101°24'52"	2344	Nianfotang of Zixishan Mountain
28	*C. reticulata* f. *simplex*	25°00'01"	101°24'60"	2333	Guzhulin of Zixishan Mountain
29	‘Ziyan’	25°05'46"	101°33'24"	1875	Lijia Village of Donggua Township
30	‘Yanzhi’	25°04'39"	101°25'03"	1950	Wangjia Mishi Temple of Yunqing village, Qianjing Township
31	‘Zibao’	25°00'47"	101°24'46"	2425	Gudelin of Zixi Mountain
32	*C. reticulata* f. *simplex*	24°43'08"	101°00'18"	1922	Damaidiwan Temple of Bajiao Township
33	‘Dalicha’	25°05'47"	101°33'24"	1886	Lijia Village of Dengguan of Donggua Township
34	‘Chuxiong-dalicha’	24°59'59"	101°25'06"	2317	Relic of Gongdelin Temple in Zixishan Mountain
35	*C. reticulata* f. *simplex*	25°00'51"	101°24'16"	2382	Songhelin of Zixishan Mountain
36	*C. reticulata* f. *simplex*	N25°00'03"	E101°24'52"	2339	Nianfotang of Zixishan Mountain
37	*C. reticulata* f. *simplex*	25°00'51"	101°24'16"	2407	Qishulin of Zixishan Mountain
38	*C. reticulata* f. *simplex*	24°59'25"	101°23'48"	1975	Mishi Temple of Hongqiang, Donghua Township
39	‘Ailaohong’	24°26'14"	101°10'26"	2230	Xinchang Vilage, Ejia Township, Shuangbai County
40	*C. reticulata* f. *simplex*	25°04'53"	101°42'05.1"	1991	Shitoumiao Mishi Temple of Cangling Village
41	‘Zilian’	24°59'55"	101°25'07"	2279	Camellia garden of Zixi Mountain
42	‘Lifang’	24°59'58"	101°25'05"	2287	Camelli garden of Zixi Mountain
43	‘Zhinan’	24°59'45.7"	101°39'23"	2314	Guzhulin Temple of Zixishan Mountain
44	‘Ziyu’	24°59'59"	101°24'56"	2288	Guzhulin Temple of Zixishan Mountain
45	‘Ziyun’	25°00'01"	101°24'58"	2303	Guzhulin Temple of Zixishan Mountain
46	‘Zidai’	25°00'02"	101°24'58"	2309	Guzhulin Temple of Zixishan Mountain
47	‘Ziwei’	24°59'58"	101°25'06"	2311	Zhiguanglin Temple of Zixishan Mountain
48	‘Zijuan’	24°59'57"	101°24'15"	2310	Relic of Gongdelin Temple of Zixishan Mountain
49	‘Zilin’	24°59'58"	101°25'02"	2307	Relic of Gongdelin Temple of Zixishan Mountain
50	‘Ziqiang’	24°59'57"	101°25'04"	2297	Relic of Gongdelin Temple of Zixishan Mountain
51	‘Zidie’	24°59'57"	101°25'04"	2296	Camelli garden of Zixi Mountain
52	‘Meigehong’	24°59'57"	101°25'02"	2299	Camelli garden of Zixi Mountain
53	‘Ziting’	24°59'57"	101°25'10"	2312	Relic of Gongdelin Temple of Zixishan Mountain
54	‘08zhichun’	24°59'57"	101°25'13"	2274	Donglin Temple of Zixishan Mountain
55	‘Chuxiong-dalicha’	24°38'49"	101°22'44"	1910	Tanshan of Zheli Village, Dajidi Township
56	‘Zifen’	25°00'20"	101°25'14"	2442	Ziding Temple of Zixishan Mountain
57	*C. reticulata* f. *simplex*	24°49'29"	101°06'44"	1940	Xiajiacun Village, Zhongshan Township
58	‘Dalicha’	24°59'55"	101°30'52"	1896	Lijia of Zhongben Village, Lucheng Township

#### Nature-based religions

Yunnan is the largest province with diversified cultures in China. There are 25 ethnic minorities native to the province, occupying 45 % of the nation’s ethnic groups. Most of the local people believe in animism religion or nature-based gods. Before the emergence of Taoism, and the entry into China of Buddhism, the original religion in Yunnan was polytheism [12, 36, 37]. Local religious beliefs, as the main way to spiritual activities in early societies gradually formed a unique aesthetic standard [37–40] affecting the aesthetic value and conservation of *C. reticulata*.

The Yi ethnic group believed in holy trees or holy forests from ancient legends, and they venerated the camellia as a holy flower. Mishi in Yi language, or ‘lord of the earth’, refers to a small temple to worship local gods, which is the Yi’s most important deity. Every year when the Yi people performed the ceremony to worship Mishi, they firstly pray to the camellia trees, and then offerup twigs of the camellia tree to the Mishi. They believed that Mishi would bless them with happiness and good fortune. Moreover, the ‘Prayer of the Dragon’ recited by Bimo, the priest of Yi people, says ‘God from the heaven dispersed three handfuls of seeds in the world, from which camellias grew and flowered all over the hillsides, thus we used the camellia to worship the god and our ancestors’. In every spring festival the Yi people decorated pine branches with camellia flowers in their courtyards as a holy tree and called it ‘tree of earth and heaven’. In Chuxiong, the old *Camellia* trees can be divided into cultivated types and wild types. Based on our investigations, the trees in the villages, temple yards and relics of temples belonged to the cultivated types. Those distributed in the wild or near the villages were wild types. Of the 58 old camellia trees cultivated within the Chuxiong Yi Autonomous Prefecture, around 10 plants were found from Mishi temples, accounting for one sixth of all old camellia trees in the area. It is an important characteristic that many old camellia trees were conserved in Mishi temples, exemplifying the conservation effect of the Yi people’s culture for this tree species.

In Lijiang, the Naxi ethnic group, like many indigenous groups in Yunnan, have a long history and traditional knowledge of growing food and medicinal plants in homegardens to support their livelihoods [41]. Historically, the Naxi relied on an indigenous system to treat health conditions primarily through consultation with local shaman priests known as Dongba (Dto’mba) as well as through herbal healers and self-care [42–44]. The Dongba believed in sacred sites, where holy forests were worshiped, and all living things were protected. These ecological and cultural important spaces, used for the transmission and preservation of ethnomedicinal knowledge that support community wellbeing and livelihoods, are at risk due to current rapid socio-economic, policy, land use and environmental changes in China [42].

### Long history and cultural connotations of *Camellia reticulata*

*Cha-hua* was cultivated in China as early as the Sui and Tang Dynasty, over 1500 years ago. The ancient selected forms, particularly with large, double or semi-double flowers, have been propagated for hundreds of years as garden plants. Some extant cultivars dated back to the Ming Dynasty (1368–1644 C.E.) [8]. In AD 898, in the drawn ‘Nanzhao Figure Biography’, in the first scroll painting named “in King’s Garden”, there were two tall trees, called ‘orange flower’ and ‘good omen flower’. From the linguistics, morphology, flower type, and Nanzhao origin place, these two trees were *C. reticulata,* and estimated to be 200 years old.

A book on history of Yunnan Province published in sixteenth century by Xie Zhaozhe (AD 1573–1620) of Ming Dynasty indicated that the *C. reticulata* was the best under the heaven. Xie also described 72 cultivars of *cha-hua* in this book. Deng Mei composed a poem of two hundreds lines in which he pointed out the ten excellences of *C. reticulata.* Zhao wrote a genealogical record of *C. reticulata* listed with nearly one hundred types. A book written by Fang Shumei in 1920 was historically important in the studies of cultivated *C. reticulata* in Yunnan Province, in which 122 poems of Ming and Qing dynasties were collected.

### Traditional cultures

#### The most popular species

More than 500 cultivars and hybrids of *C. reticulata* have been recognized [17]. However, only dozens of improved varieties are common. The most popular ones are the traditional top cultivars (Table 4).

**Table 4:** Top traditional cultivars of *Camellia reticulata*

	Cultivar names	Flower type	Flower color	Blooming period	Remarks
1	‘Dwarf Rose’	Rose double	Peach blossom	Feb–Apr	One of the eight famous cultivars in Dali
2	‘Baby Face’	Rose double	Pinkish white	Mar–Apr	One of the shallowest color cultivars
3	‘Purple Gown’	Peony double	Prune	Feb–Mar	One of the darkest color cultivars
4	‘Dali Camellia’	Peony double	Red	Jan–Mar	
5	‘Pine Cone Scale’	Rose double	Red	Jan–Mar	
6	‘Peony Camellia’	Peony double	Peach pink	Feb–Mar	Later blooming.
7	‘Large carnelian’	Peony double	Multi-color	Jan–Mar	Local name: pork blood mix tofu
8	‘Chrysanthemum Petal’	Rose double	Pink	Dec–Mar	A very popular one
9	‘Reticulate Leaf Spinel Pink’	Semi-double	Sliver red	Feb–Apr	
10	‘Thick Leaf Butter Wing’	Semi-double	Red	Jan–Apr	
11	‘Tsingan Camellia’	Peony double	Red	Feb–Mar	
12	‘Guomei Camellia’	Semi-double	Red	Jan–Mar	In memory of a famous botanist, Prof. Guomei Feng
13	‘Lion’s Head’	Peony double	Prune	Jan–Mar	Local name: nine stamens eighteen petals
14	‘Early Crimson’	Semi-double	Peach blossom	Dec–Mar	The earliest blooming one
15	‘King Peony’	Peony double	Peach blossom	Oct–Feb	Maximum number of petals
16	‘Reticulate Leaf Crimson’	Semi-double	Red	Feb–Apr	
17	‘Treasure Pearl Camellia’	Peony double	Red	Feb–Mar	Ancient *Camellia* cultivar
18	‘Willow Leaf Spinel Pink’	Semi-double	Slivery red		

#### Regional features

Different regions have different cultural atmospheres. People of different ethnic groups with their own traditional culture have enriched the diversity and cultural values of camellia. Dali has rich variety resources of *Camellia*. The Bai people in Dali promoted *Camellia* as the ‘King of Flowers’, and during the annual Lunar New Year from February ninth to fifteenth it was the time of the ‘worship flower fair’. In Dali, every family grows *Camellia* in their home yard. *C. reticulata* trees of hundreds years old can be found. More than that, *Camellia* is a symbol of Dali. In ancient times, *Camellia* was the symbol of nobility. Some varieties like ‘Lion’s Head’, ‘Red Gown’ (Gown means official’s robe), ‘Large Carnelian’, are all precious cultivars only nobility and gentry could hold. Nowadays, *C. reticulata* is not only an excellent ornamental flowering plant, but also a precious gift for friends.

**Fig. 4:**
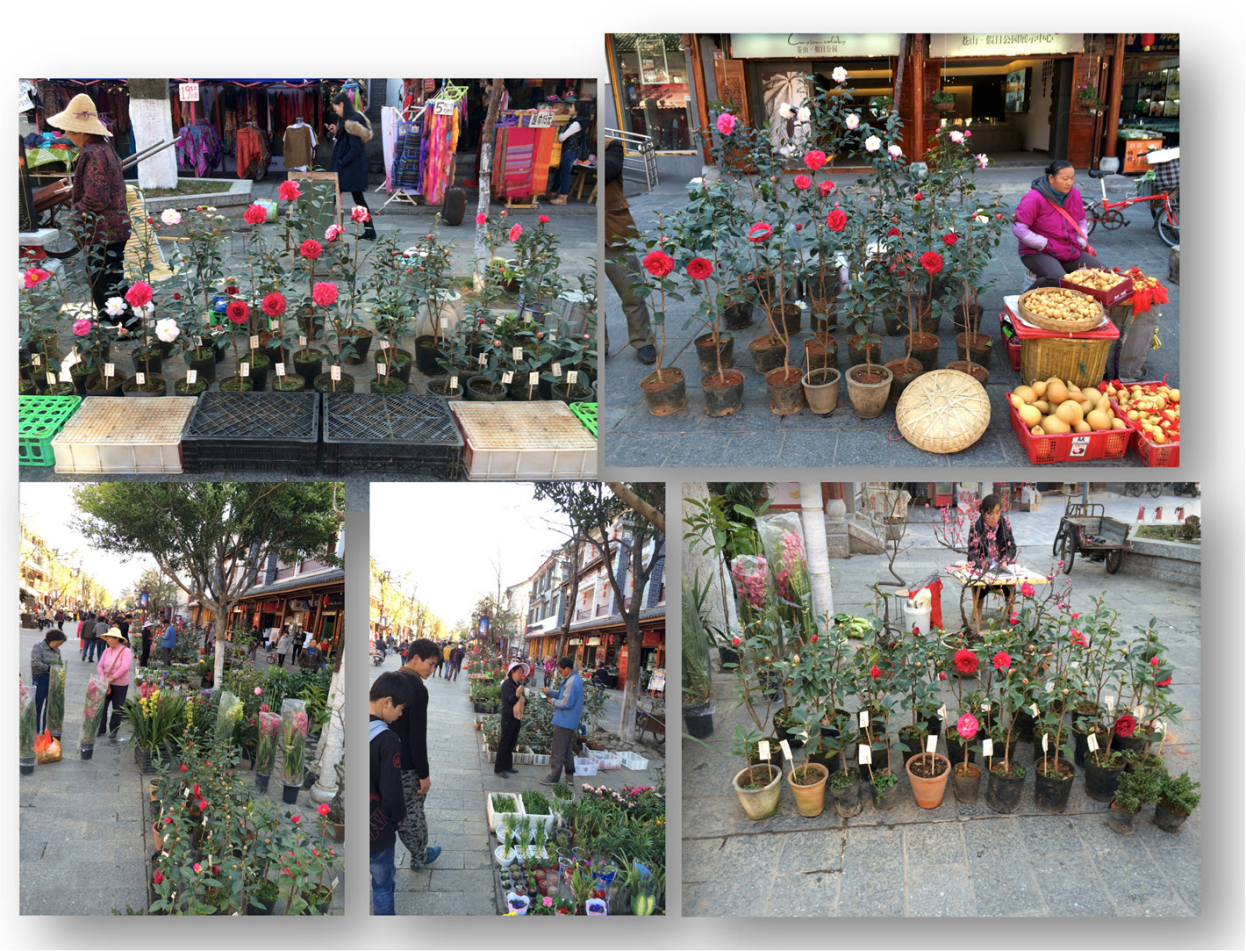
The *Camellia* market in the Old Town, Dali

In our surveys, the most preferred cultivars are also the traditional ones. On every weekend, there is the *Cha-hua* Market in the old town of Dali (Fig. 4). In the market the price varied from 40 CNY (Chinese yuan) to 300 CNY (ca. 1USD = 6.5 CNY) per seedling. In the season of Spring Festival from December to February, millions of *Camellia* trees bloom in and around the old town, ancient alleys and yards.

**Fig. 5:**
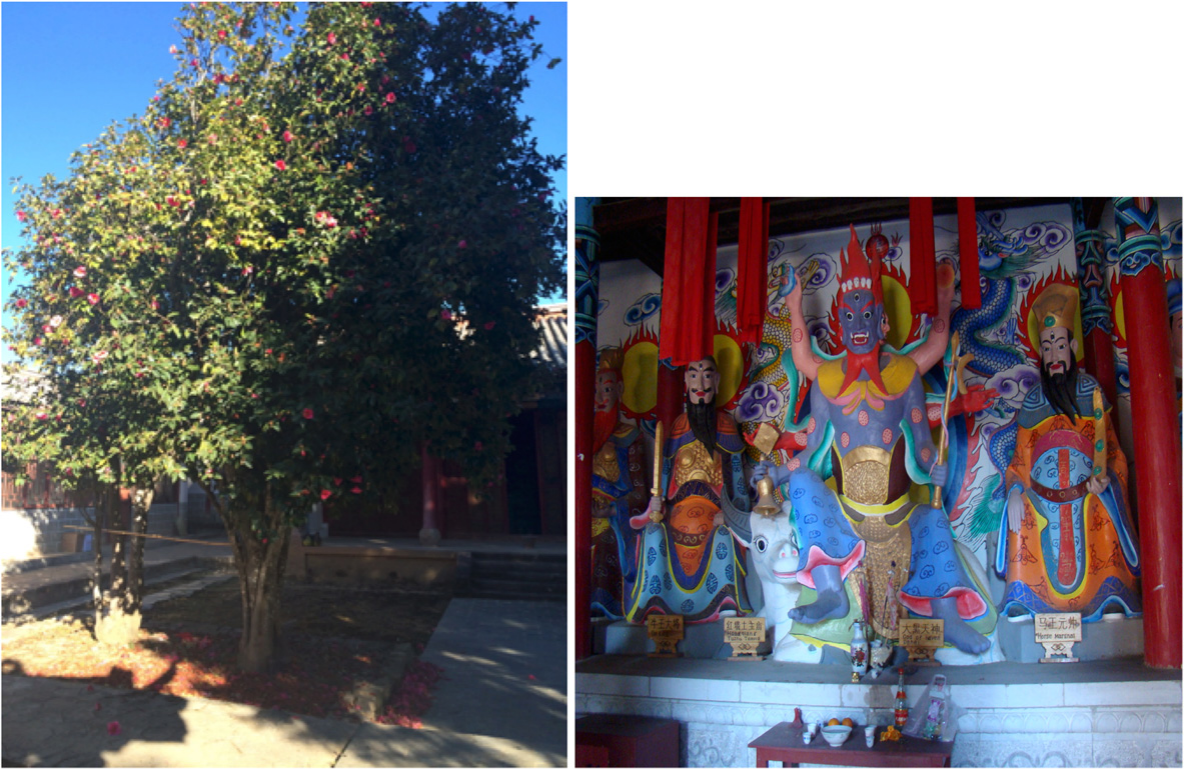
Offering *Camellia reticulata* to Mishi in the temple

In the Zixi Mountains area of Chuxiong, the widely distributed native *C. reticulata* trees can be found. The residents of this area are mainly Yi, Miao and Han nationalities. The Yi people honor *cha-hua* as a holy flower as a sacrifice to heaven and ancestors, and prohibit the climbing of camellia trees or breaking their branches. In the Mishi temple of every village, Yi people plant *cha-hua* to enjoy their beauty and as a sacrifice to the Mishi (Gods) (Fig. 5). Protecting the camellia trees for this role in local religious activities has served to protect most of ancient camellia trees in Zixi Mountains area.

The Bai and Han nationalities regarded the camellia as a tree that focuses good fortune. They believe the tree can gather aura, and straighten out *Fengshui* (a form of geomancy). For the Bai and Han nationalities the camellias were planted predominantly in gardens and in family ancestral temples. About 860 years ago, during the Dali Kingdom period, the Prime Minister Gao Liangchen and his wife abdicated to Weixi Mountain, which became their fief where they had lived in seclusion since 1150. They built their castle on the mountain (know as ‘Prime Minister’s House in the Mountain’, or Shi Sangchen) together with a Buddhism temple. It is the first known record of the cultivation of camellia trees in the region. From 200–500 years to the Ming and Qing dynasties (1368–1855), Zixi Mountains became a sacred Buddhist site, with nearly 100 temples, nunneries, and sacred groves. There are many camellia cultivars growing on temple’s relics, including the cultivars: ‘Zixi’, ‘East Lin’, ‘West Lin’, ‘Dali’, ‘Zibao’, ‘Songzike’, ‘Baby Face’, ‘Luchengchun’. The ancient *Camellia* trees are found in the temples and on the relic site of buildings, although these places were destroyed in the war in 1856–1872. In this area, hybridization of the artificially cultivated camellia in the area with the wild *C. reticulata* (with single petals) has frequently occurred resulting enriching the diversity of *C. reticulata*. The Zixi Mountains of Chuxiong created a center of *cha-hua* natural variation.

**Fig. 6:**
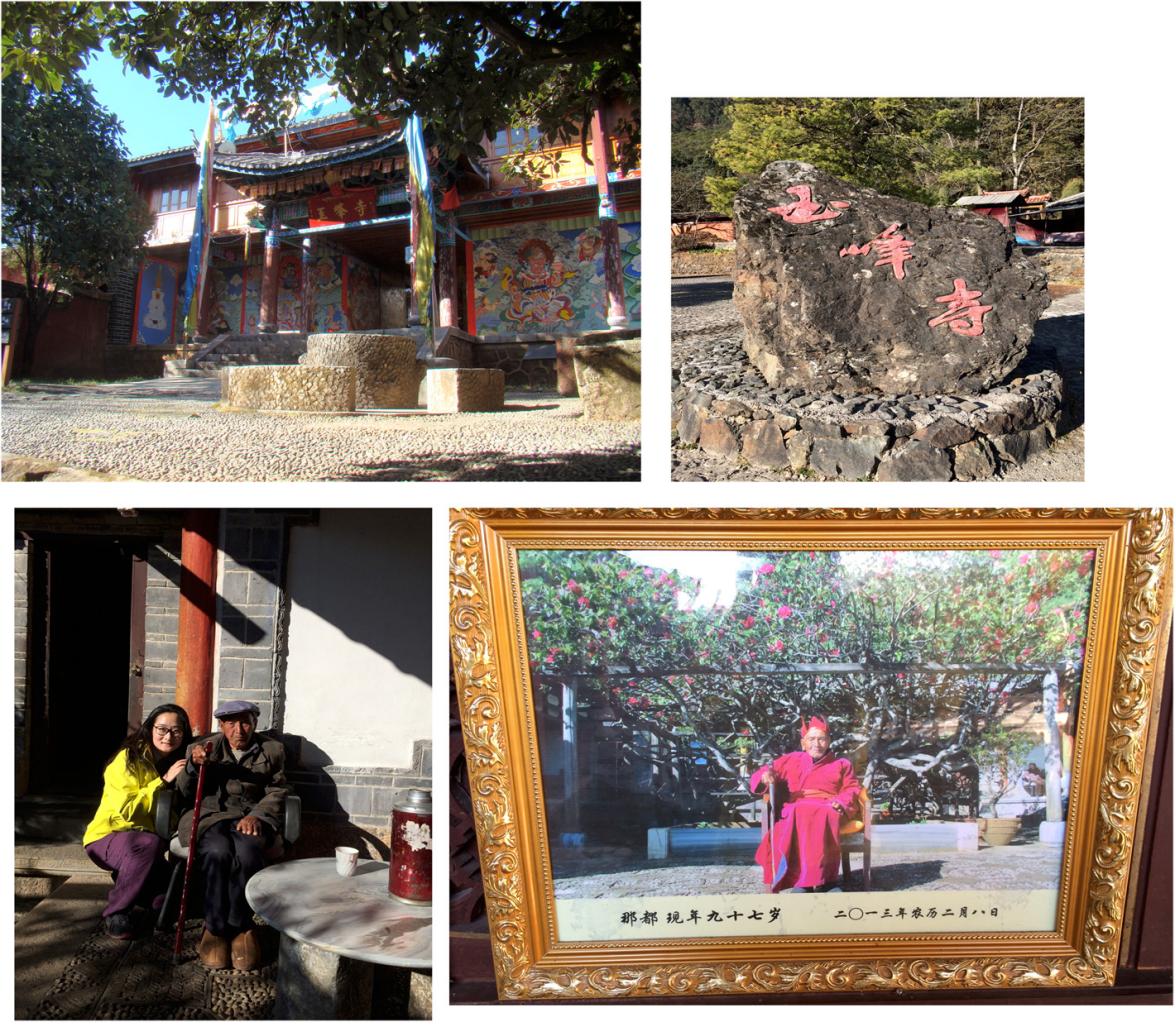
‘King of *Camellia*’ (‘Shizitou’ and *C. reticulata* f. *simplex*) of Yunfeng Temple in Lijiang, and its guards Nadu Lama, an old Naxi people

The Yufeng Temple in Lijiang, located in the south of Yulong Snow Mountains is famous for its *cha-hua* tree named “Thousands of Camellia Flower”. The Yufeng Lamasery was built at the end of Qing Dynasty, which is one of the five well-known lamaseries in Lijiang. The yard of main hall of the temple was built in the architectural style of the Qing Dynasty with the traditional Chinese courtyard design, a combination of Tibetan Buddhism and Han Buddhism architectural styles. This famous *Camellia* tree was planted in the year of Chenghua, Ming Dynasty (around AD 1465–1487) in the northwest garden to the main hall. Two branches called “happiness trees” twisted to make a main trunk. In the spring season, the camellia tree blossoms are in full splendor, and this tree has been honored by the name of the King of the *Camellia*. An old Naxi man, Nadu Lama, has guarded this precious tree his entire life (Fig. 6).

#### Potential cultural significance

##### Cultivars named for good fortune

Chinese names for most *C. reticulata* cultivars are according to the morphological characteristics of their respective flowers. Most of their Chinese names have meanings that imply good luck. Different colors of petals represent different meanings. ‘purple gown’ is a very popular and traditional variety with prune color. A family with politicians or businessmen in their family will grow this cultivar to bless them to be successful in official careers or business ventures. Other examples of cultivar names with positive means are ‘Jade Belt Purple Gown’, ‘Vermilion Purple Gown’, and‘Red Splendid Gown’. Some *Camellia* cultivar names are related to the Buddhism, for example ‘Buddha Lotus’, which means the flower morphology of this cultivar is similar to lotus, the Buddhist flower.

##### Allusions

The cultivar ‘Lion’s Head’, comes from the famous Novel of Octave, where Devas and Nagas mentioned a cultivar with ‘nine stamens and eighteen petals’. The cultivar ‘Mi Yi Lu’ was adopted from a Yi girl named Miyilu who comes from the most beautiful love story in Yi communities. ‘Guomei *Camellia*’ is a cultivar in memory of Professor Guomei Feng, a famous botanist who devoted himself to study *Camellia* for many years.

### Impact from Southwest Silk Road

The old *Camellia* trees of cultivated type were mainly distributed in the villages and temples in Kunming, Dali, Chuxiong, Fengqing, and Tengchong along the old Southwest Silk Road. This old road promoted not only the development of commodity circulation and trade, but also the cultural exchange including religion and humanity, especially the Buddhism, of of which the cultivation of the camellia tree was closely linked.

## Conclusion

As one of the most popular ornamental flowers in China, *cha-hua* or *Camellia reticulata* much attention has been paid to its commercial cultivation and breeding. The conservation and use by traditional cultures of *C. reticulata* has been predominantly ignored. This paper studied the influence of traditional culture on the introduction and domestication of wild *Camellia* species, breeding and selection of different varieties, and their dissemination in Yunnan Province.

The process of *C. reticulata* introduction and domestication has relied on local different ethnic groups and their traditional beliefs and practices, which has been recorded in a great number of historical documents. The ancient *Camellia* trees continue to be protected in the yards of old temples and other historical sites. *Cha-hua* culture has penetrated into many components of the social lives and ethnic communities. Yunnan people are proud of this valuable diversity of *cha-hua* and continue to protect it and use it in the tradition culture of their daily lives.

## Consent

Permissions were provided by all participants in this study and with the nationalities interviewed for this study. Consent was obtained from the participants prior to this study being carried out. Nadu Lama declared that he has no objection to the publication of his pictures (Fig. 5) in the journal. The authors have all copyrights.
